# Emerging roles of circRNA_001569 targeting miR-145 in the proliferation and invasion of colorectal cancer

**DOI:** 10.18632/oncotarget.8589

**Published:** 2016-04-05

**Authors:** Huijun Xie, Xiaoli Ren, Sainan Xin, Xiaoliang Lan, Guifeng Lu, Yuan Lin, Shaoshan Yang, Zhicheng Zeng, Wenting Liao, Yan-Qing Ding, Li Liang

**Affiliations:** ^1^ Department of Pathology, Nanfang Hospital, Guangzhou, Guangdong, China; ^2^ Department of Pathology, School of Basic Medical Sciences, Southern Medical University, Guangzhou, Guangdong, China; ^3^ Guangdong Province Key Laboratory of Molecular Tumor Pathology, Guangzhou, Guangdong, China; ^4^ Department of General Surgery, Nanfang Hospital, Guangzhou, Guangdong, China

**Keywords:** hsa_circ_001569, miR-145, colorectal cancer, FMNL2, BAG4

## Abstract

Circular RNAs (circRNAs), a large class of RNAs, have recently shown huge capabilities as gene regulators in mammals. Some of them bind with microRNAs (miRNAs) and act as natural miRNA sponges to inhibit related miRNAs’ activities. Here we showed that hsa_circ_001569 acted as a positive regulator in cell proliferation and invasion of colorectal cancer (CRC). Moreover, hsa_circ_001569 was identified as a sponge of miR-145 and up-regulated miR-145 functional targets E2F5, BAG4 and FMNL2. In CRC tissues, circ_001569 negatively correlated with miR-145, and miR-145 correlated negatively with E2F5, BAG4 and FMNL2 expressions. Our study reveals a novel regulatory mechanism of circ_001569 in cell proliferation and invasion in CRC, provides a comprehensive landscape of circ_001569 that will facilitate further biomarker discoveries in the progression of CRC.

## INTRODUCTION

Colorectal cancer (CRC) is a major health problem representing the third most common malignancy and the fourth most common cause of death in the world [1]. Thus, it is essential to identify some new molecular markers to raise the efficiency of tumor diagnosis and to predict prognosis of the patients or even for therapeutic application.

Circular RNAs (circRNAs), a class of endogenous RNAs mainly composing of transcript from exons which are formed by non-colinear reverse splicing, are widely expressed in human cells and play important roles in the regulation of gene expression at post-transcriptional level [2, 3]. Several circRNAs regulate gene expression acting as competing endogenous RNA (ceRNAs) [4], also known as microRNAs (miRNAs) sponge. MiRNA activity is affected by the presence of miRNA sponges transcripts [5–7]. CircRNAs sequester miRNAs to terminate regulation of their target genes [8, 9]. Further study on circRNAs will enable us to better understand the pathological mechanisms and improve the prevention and diagnosis of the associated diseases.

In comparison to circRNAs, miRNAs are extremely well studied. Large evidence has shown that miRNAs are involved in cancer as tumor suppressors or oncogenes, thereby being also potential cancer biomarkers [10]. Many miRNAs have been reported to be closely related to the progression of CRC [11–14]. MiR-145 was identified as a tumor suppressor in prostate cancer, renal cell cancer, etc [15–17]. In 2003, Michael et al. illustrated tumor suppressor-like activities for miR-143 and miR-145 in colon cancer and hypothesized that these miRNAs were targeting ERK5 and IRS1 [10]. MiR-145 has been associated with patient's survival after diagnosis with CRC [18, 19]. MiR-145 has potential clinical importance given its ability to predict survival of CRC patients with 81% accuracy [20]. However, the function and regulatory mechanisms of miR-145 in the progression of CRC need to be further investigated.

In this study, we provide evidences that circ_001569 promotes cell proliferation and invasion in CRC. Mechanically, circ_001569 acts as a miRNA sponge to directly inhibit miR-145, and subsequently up-regulates miR-145 targets E2F5, BAG4 and FMNL2 to exert its tumor promoting function in CRC cells.

## RESULTS

### Circ_001569 is up-regulated in CRC tissues and correlates with aggressive characteristics of CRC

**Figure 1:**
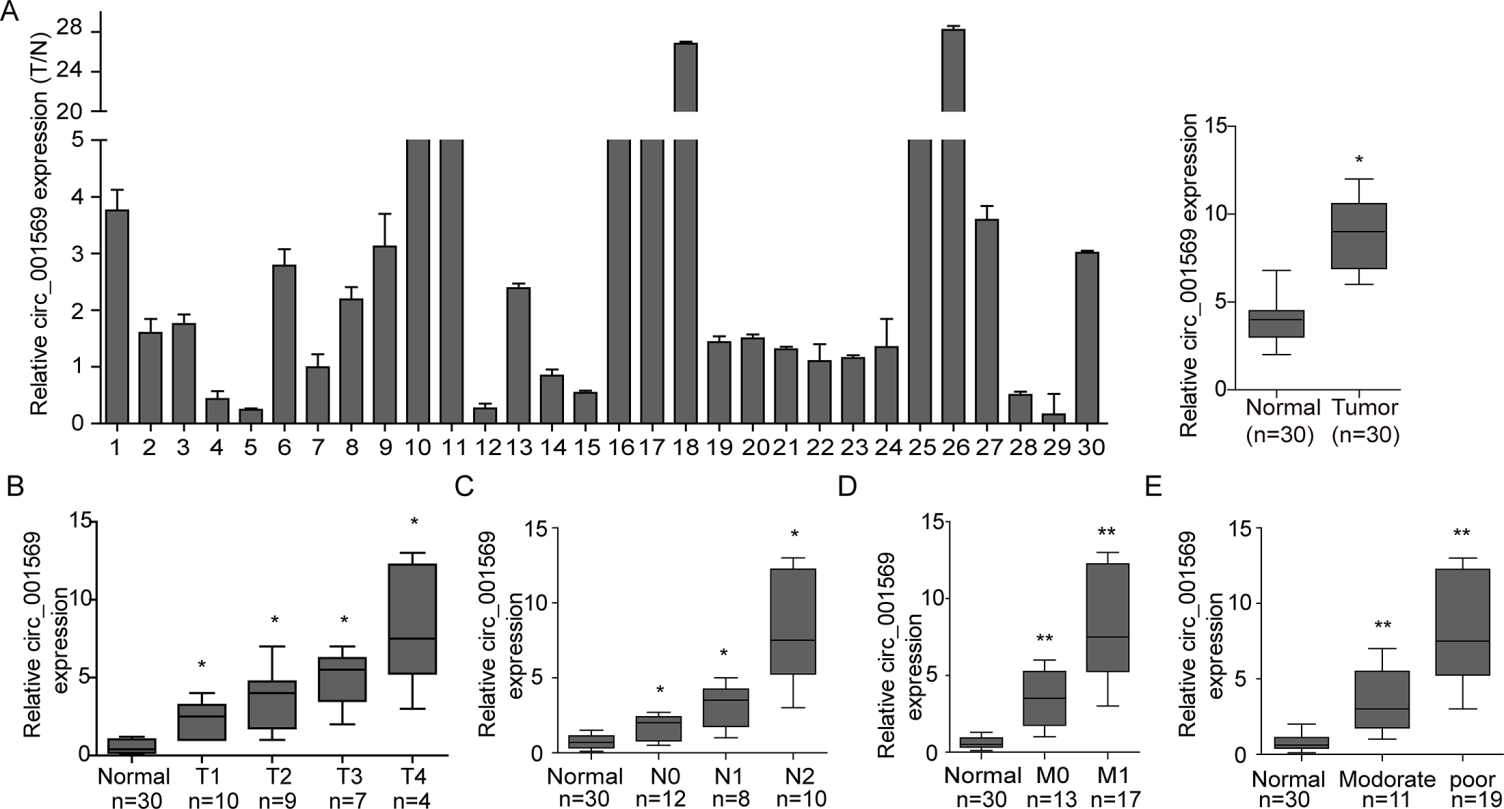
Up-regulation of circ_001569 correlates with aggressive characteristics of CRC (**A**) Expression of circ_001569 in the 30 paired human CRC tissues and normal intestine epithelial tissues by real-time PCR. Each bar represents the mean of 3 independent experiments. (**B**) Correlation between circ_001569 expression and T classification (T1–T4) in 30 cases of CRC tissues and normal intestine epithelial tissues. (**C**) Correlation between circ_001569 expression and N classification of CRC (N0–N2). (**D**) Correlation between circ_001569 expression and distant metastasis. (**E**) Correlation between circ_001569 expression and differentiation of CRC. *p < 0.05, **p < 0.01.

Based on the previous study that 85 circular RNAs were found in twelve matched normal and CRC samples [21], and the results that we profiled circ2Traits [22] database to explore circRNAs which was associated with CRC, we selected has_circ_001569 as a potential regulator in CRC progression. To explore the expression pattern of circ_001569 in CRC tissues, we detected the expression of circ_001569 by real-time PCR analysis in 30 paired samples of CRC patients. The result revealed that the level of circ_001569 was significantly higher in the CRC tissues than adjacent normal tissues (*p* < 0.05, Figure 1A). Circ_001569 expression level closely correlated with differentiation and TNM classification (Table 1). Circ_001569 expression level was increased along with the progression of T classifications (Figure 1B, *p* < 0.05), N classifications (Figure 1C, *p* < 0.05), distant metastasis (Figure 1D, *p* < 0.01) and poor differentiation (Figure 1E, *p* < 0.01). The results suggest a link between up-regulation of circ_001569 and aggressive characteristics of CRC.

**Table 1:** Correlation between clinicopathological features and circ_001569 expression in 30 cases of CRC tissues

	circ_001569 expression		
Characteristics	Low	High	*P*
Age			
≤ Mean (57)	9	7	0.700
> Mean (57)	8	6	
Gender			
Male	7	9	0.800
Female	5	9	
Differentiation			
Well-moderate	7	4	0.000
Poor	4	15	
T classification			
1–2	12	7	0.024
3–4	2	9	
N classification			
0	4	8	0.041
1–2	3	15	
Distant metastasis			
No	7	6	0.000
Yes	6	11	

### Circ_001569 promotes the proliferation and invasion of CRC cells

**Figure 2:**
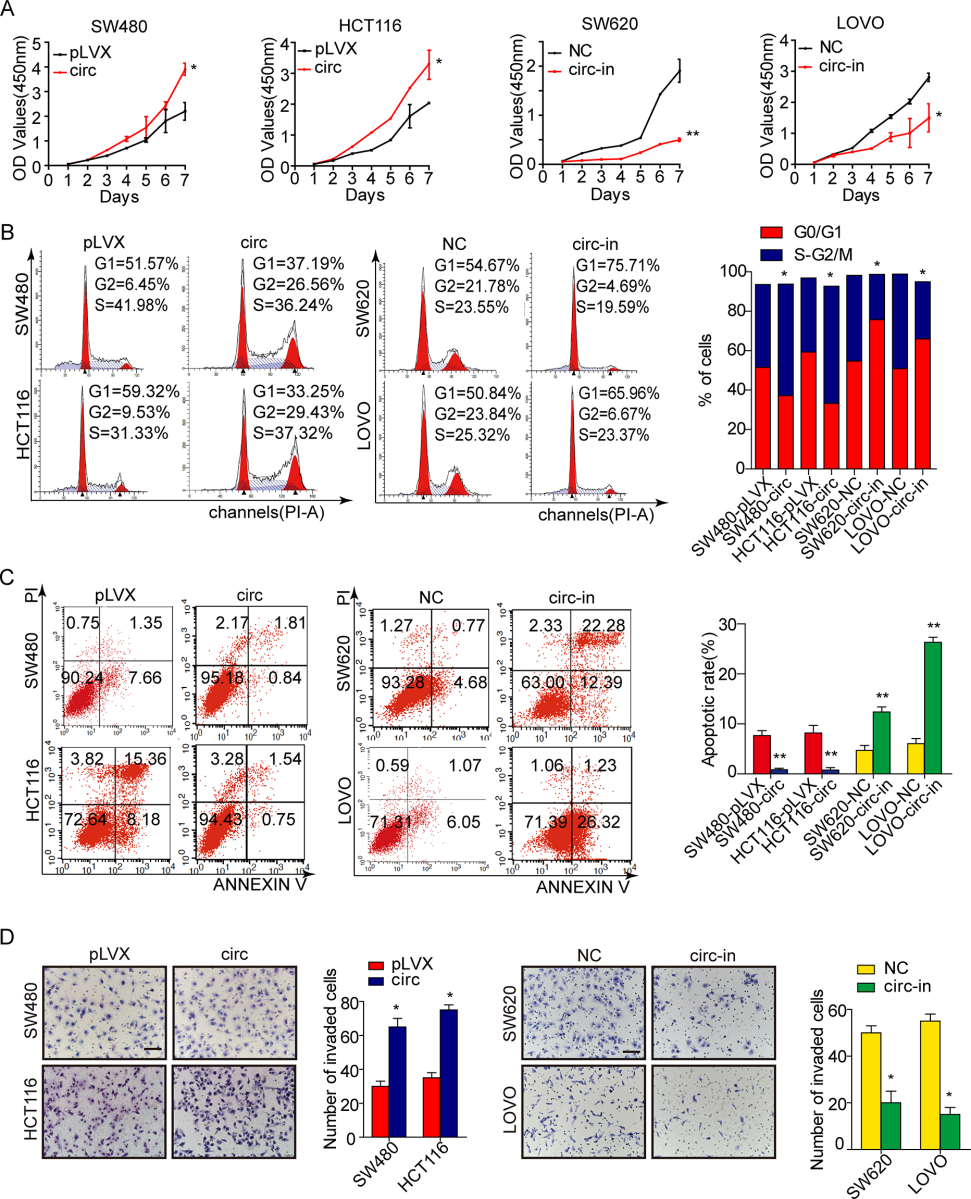
Circ_001569 promotes proliferation and tumor growth of CRC cells (**A**) Effect of circ_001569 on cell proliferation *in vitro* by CCK8 assay. (**B**) Effect of circ_001569 on CRC cell cycle by flow cytometry. (**C**) Effect of circ_001569 on CRC cell apoptosis by flow cytometry. (**D**) Effect of circ_001569 on CRC cell invasion *in vitro* by Boyden chamber. Morphological comparison of cells penetrating the artificial basement membrane was also shown. Scale bars represent 50 μm. Error bars represent mean ± SD from three independent experiments. *p < 0.05, **p < 0.01.

To detect the function of circ_001569 in the progression of CRC, we performed gain-of function and loss-of-function assays. According to endogenous expression of circ_001569 in 6 CRC cell lines (Figure S1A), we over-expressed circ_001569 in SW480 and HCT116 cell lines and silenced circ_001569 in SW620 and LOVO cells (*p* < 0.01, Figure S1B). Then we assessed the effect of circ_001569 on the proliferation of CRC cells. Results of CCK-8 assays showed that the increased proliferative ability was observed in circ_001569 expressing cells, while a sharp reduction in the proliferation rate was shown in circ_001569 depleting cells (*p* < 0.05, Figure 2A). To explore the possible mechanism of circ_001569′s function in controlling CRC cell proliferation, we determined the distribution of cells within the stages of the cell cycle by flow cytometry. The cells treated with circ_001569 showed a significant decrease in the percentage of cells in the G1/G0 peak and a increase in the percentage of cells in the S and G2/M peak, while circ_001569 inhibitor showed the opposite effects (*p* < 0.05, Figure 2B). After that, we measured the effect of circ_001569 on apoptosis of CRC cells. The results showed that the rate of apoptosis was significantly lower in circ_001569 expressing SW480 and HCT116 cells. However, the rate of apoptosis was remarkably increased when circ_001569 was inhibited in SW620 and LOVO cells (*p* < 0.01, Figure 2C). These results demonstrate that circ_001569 promotes CRC cell proliferation, at least partly by eliminating cell cycle arrest and inhibiting apoptosis. We also analyzed the effect of circ_001569 on the invasion of CRC cells. Results of Boyden chamber assays showed that over-expression of circ_001569 in SW480 and HCT116 cells effectively increased the invasive abilities, while suppression of circ_001569 in SW620 and LOVO cells had the reverse effect (*p* < 0.05, Figure 2D). These above data make it clear that circ_001569 promotes cell proliferation and invasion in CRC.

### Circ_001569 inhibits the transcription activity of miR-145 and up-regulates miR-145 targets E2F5, BAG4 and FMNL2

**Figure 3:**
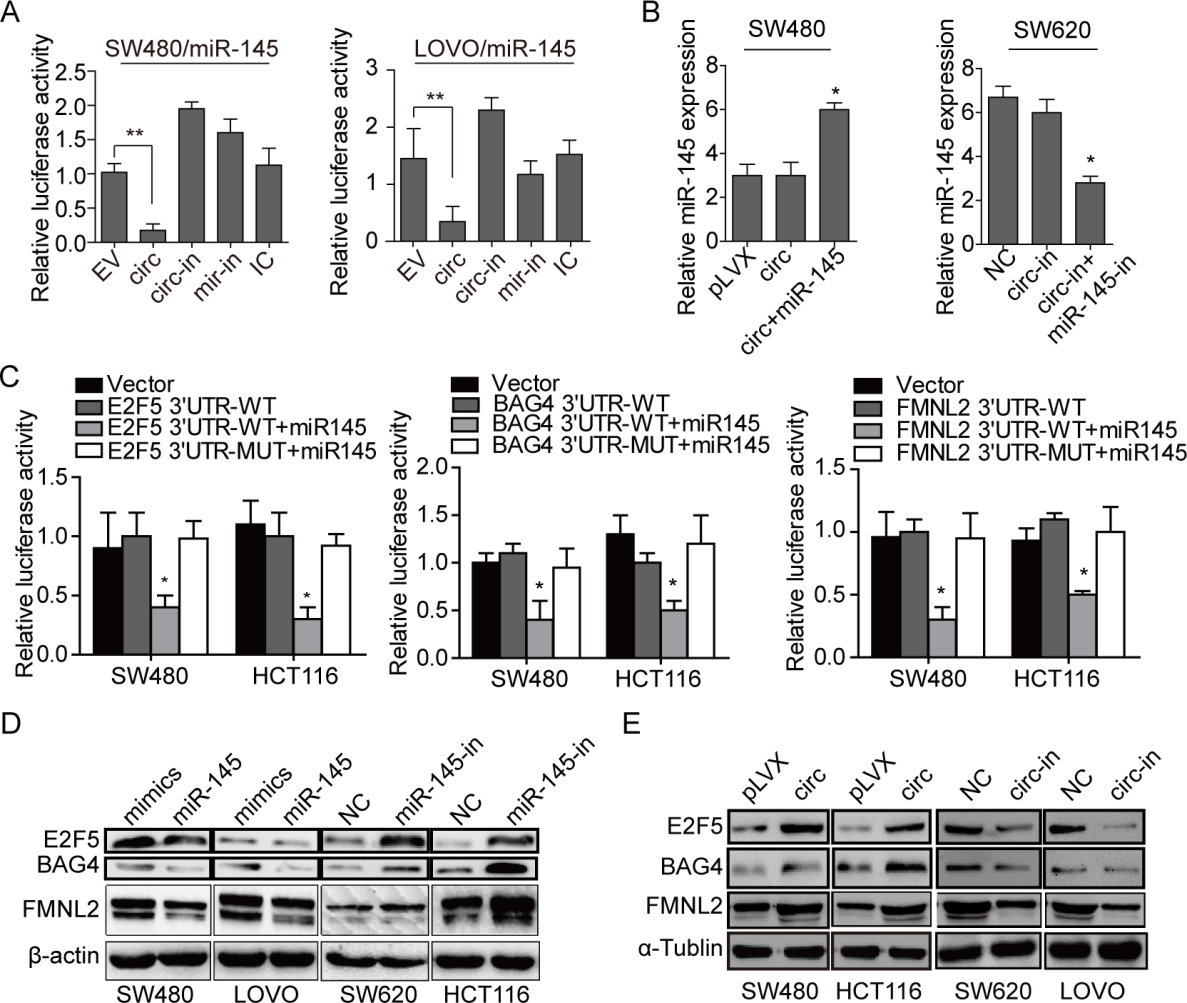
Circ_001569 inhibits the transcription activity of miR-145 and up-regulates miR-145 targets E2F5, BAG4 and FMNL2 (**A**) Effects of empty vector (EV), circ_001569, circ_001569 inhibitor (circ-in), miR-145 inhibitor (miR-in) and miRNA inhibitor control (IC) on the luciferase activity of miR-145 in SW480 and LOVO cells by luciferase reporter assay. (**B**) Expression of miR-145 in cells transfected with circ_001569, circ_001569/miR-145, circ_001569 inhibitor (circ-in) and circ-in/miR-145 inhibitor (miR-145-in), respectively. (**C**) Effect of miR-145 on the luciferase activities of E2F5, BAG4 and FMNL2 3′UTR and their mutant fragments in SW480 and HCT116 cells by luciferase reporter assay. (**D**) Expressions of E2F5, BAG4 or FMNL2 in miR-145 expressing or depleting cells by Western Blotting and was normalized by β-actin expression. (**E**) Expressions of E2F5, BAG4 or FMNL2 in circ_001569 expressing or depleting cells by Western Blotting and was normalized by α-tublin expression. Error bars represent mean ±SD from three independent experiments. *p < 0.05, **p < 0.01.

Evidences have shown that CircRNAs sequester miRNAs to terminate regulation of their target genes [8, 9], we thus speculated that circ_001569 could target miRNAs to inhibit their expression. In order to screen miRNAs which could be combined with circ_001569, we profiled two public databases (StarBase v2.0 [23] and circBase [24]), and found that miR-145 had a binding site of circ_001569 (Figure S1C), which was predicted by both databases. Indeed, reduction of luciferase activity was observed upon the combination of circ_001569 and miR-145 (*p* < 0.01, Figure 3A). Interestingly, circ_001569 did not affect the expression of miR-145 (Figure 3B).

Given that miRNAs exert their functions through regulating the expression of their target genes, three common bioinformatic algorithms (TargetScan, Pictar and miRANDA) were used to predict the mRNA targets of miR-145. Based on the representation of miR-145 sites in their 3′UTRs, > 200 mRNAs were predicted to be regulated by miR-145. Among those mRNAs, E2F5, BAG4 and FMNL2 were predicted by all three databases. We cloned the 3′UTR fragments of E2F5, BAG4 and FMNL2 containing miR-145 binding sites and their mutant fragments (Figure S1D) into the pGL3-basicluciferase reporter vectors. A consistent reduction of luciferase activity was observed upon miR-145 transfection in both CRC lines, but mutations in the tentative miR-145-binding seed region in E2F5, BAG4 and FMNL2 3′ UTRs abrogated the suppressive effect (Figure 3C). These results demonstrate that miR-145 can directly target E2F5, BAG4 and FMNL2 in CRC cells by interacting with the 3′UTRs of these genes. In addition, ectopic miR-145 in SW480 and LOVO cells reduced the levels of E2F5, BAG4 and FMNL2, while knockdown of miR-145 in SW620 and HCT116 cells led to increased expressions of E2F5, BAG4 and FMNL2 (Figure 3D). After that, we explored whether circ_001569 regulated the expression of miR-145 targets. Results of Western Blotting showed that circ_001569 increased the protein levels of E2F5, BAG4 and FMNL2 in SW480 and HCT116 cells while knockdown of circ_001569 in SW620 and LOVO cells had the reverse effects (Figure 3E). These results confirm that as a miR-145′s sponge, circ_001569 does not affect the expression of miR-145, but inhibits the transcription activity of miR-145, and subsequently up-regulates E2F5, BAG4 and FMNL2 proteins.

### Circ_001569 promotes the proliferation and invasion of CRC cells by regulating miR-145 and its targets

**Figure 4:**
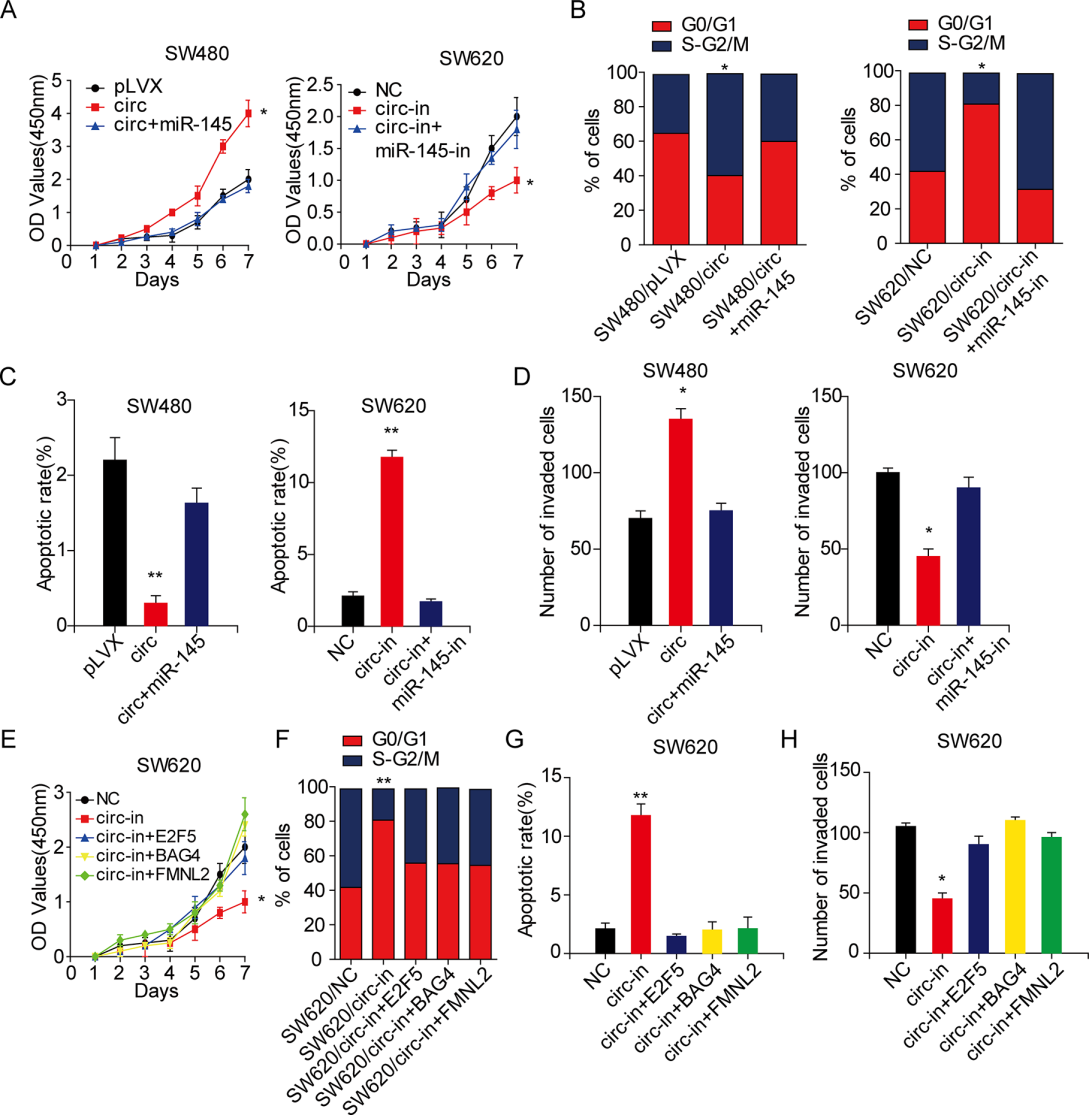
Circ_001569 promotes the proliferation and invasion of CRC cells by regulating miR-145 and its targets (**A**) Effect of miR-145 on circ_001569 induced cell proliferation by CCK-8 assay. (**B**) Effect of miR-145 on circ_001569 induced CRC cell cycle by flow cytometry. (**C**) Effect of miR-145 on circ_001569 induced CRC cell apoptosis by flow cytometry. (**D**) Effect of miR-145 on circ_001569 induced cell invasion *in vitro* by Boyden chamber. (**E**) Effect of E2F5, BAG4 or FMNL2 on circ_001569 knockdown induced cell proliferation by CCK-8 assay. (**F**) Effect of E2F5, BAG4 or FMNL2 on circ_001569 knockdown induced CRC cell cycle by flow cytometry. (**G**) Effect of E2F5, BAG4 or FMNL2 on circ_001569 knockdown induced CRC cell apoptosis by flow cytometry. (**H**) Effect of E2F5, BAG4 or FMNL2 on circ_001569 knockdown induced cell invasion *in vitro* by Boyden chamber. Error bars represent mean ± SD from three independent experiments.*p < 0.05, **p < 0.01.

To ascertain whether miR-145 was required for circ_001569 induced cell proliferation and invasion, we introduced miR-145 into circ_001569 expressing SW480 cells and miR-145 inhibitor into circ_001569 depleting SW620 cells, respectively (Figure 3B). Results of functional assays revealed that circ_001569 significantly increased the growth rate of SW480 cells, while miR-145 could rescue this effect (*p* < 0.05, Figure 4A). Suppression of circ_001569 inhibited the proliferation of SW620 cells. However, silence of miR-145 abolished the suppression induced by circ_001569 knockdown (*p* < 0.05, Figure 4A). Moreover, circ_001569 accelerated SW480 cells entering into G2/M phase and inhibited cell apoptosis, while re-introduction of miR-145 had the reverse effects (Figures 4B, S2A, 4C, S2B). Results of invasion assays also revealed that circ_001569 significantly increased the invasive ability of SW480 cells, while miR-145 could rescue this effect (*p* < 0.05, Figures 4D, S2C). Knockdown of circ_001569 inhibited the invasion of SW620 cells. However, silence of miR-145 reversed the effect of circ_001569 knockdown on cell invasion (*p* < 0.05, Figures 4D, S2C). These results support that circ_001569 promotes the proliferation and invasion of CRC cells by inhibiting miR-145.

Next, we examined whether miR-145 targets were responsible for cell behaviors induced by circ_001569. We over-expressed E2F5, BAG4 or FMNL2 in circ_001569 depleting cells (Figure S1E). As expected, constitutive expression of E2F5, BAG4 or FMNL2 remarkably abrogated circ_001569 knockdown-induced suppression on cell proliferation (Figure 4E). Moreover, introduction of E2F5, BAG4 or FMNL2 in circ_001569 depleting SW620 cells increased the percentage of cells in G2/M phase (Figures 4F, S2A) and reduced the apoptosis rate (Figures 4G, S2B). E2F5, BAG4 or FMNL2 also obviously rescued the suppression upon circ_001569 knockdown in cell invasion (Figures 4H, S2C). Collectively, these data illustrate that circ_001569 promotes the proliferation and invasion of CRC cells by inhibiting miR-145, and subsequently up-regulates the functions of miR-145 targets E2F5, BAG4 or FMNL2.

### E2F5, BAG4 and FMNL2 are functional targets of miR-145 in CRC cells

**Figure 5:**
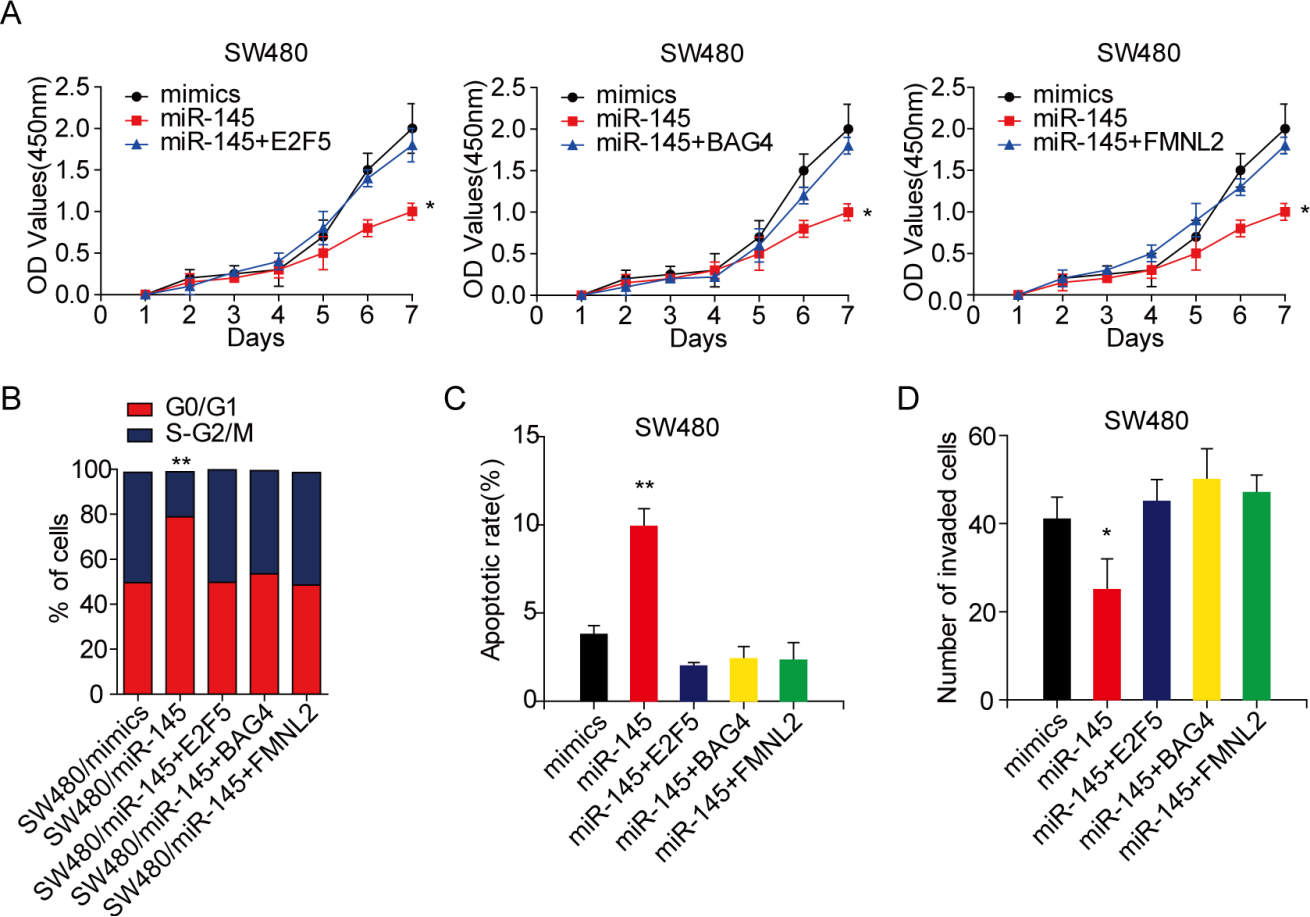
E2F5, BAG4 or FMNL2 plays crucial roles in miR-145 induced the proliferation and invasion of CRC cells (**A**) Effect of E2F5, BAG4 or FMNL2 on miR-145 induced cell proliferation by CCK-8 assay. (**B**) Effect of E2F5, BAG4 or FMNL2 on miR-145 induced CRC cell cycle by flow cytometry. (**C**) Effect of E2F5, BAG4 or FMNL2 on miR-145 induced CRC cell apoptosis by flow cytometry. (**D**) Effect of E2F5, BAG4 or FMNL2 on miR-145 induced cell invasion *in vitro* by Boyden chamber. Error bars represent mean ±SD from three independent experiments.*p < 0.05, **p < 0.01.

We performed functional assays to identify whether the miR-145 target genes were required for cell behaviors induced by miR-145. According to endogenous expression of miR-145 in 6 CRC cell lines (Figure S1F), we over-expressed miR-145 in SW480 and LOVO cell lines and silenced miR-145 in HCT116 and SW620 cells (*p* < 0.01, Figure S1G). After that, we transfected E2F5, BAG4 or FMNL2 expressing vector without 3′UTR region into miR-145 expressing SW480 cells, respectively (Figure S1H). Results showed that E2F5, BAG4 or FMNL2 remarkably rescued the suppression of cell proliferation induced by miR-145 (Figure 5A). Furthermore, ectopic E2F5, BAG4 or FMNL2 led to a rather modest increase in the G2/M phase (Figures 5B, S2D) and a decrease in apoptosis in miR-145 expressing cells (Figures 5C, S2E). Restoring E2F5, BAG4 or FMNL2 expression also significantly abolished miR-145 induced suppression on cell invasion (Figures 5D, S2F). These data make it obvious that miR-145 inhibits proliferation and invasion by targeting E2F5, BAG4 or FMNL2.

### MiR-145 negatively correlates with circ_001569 or its targets

**Figure 6:**
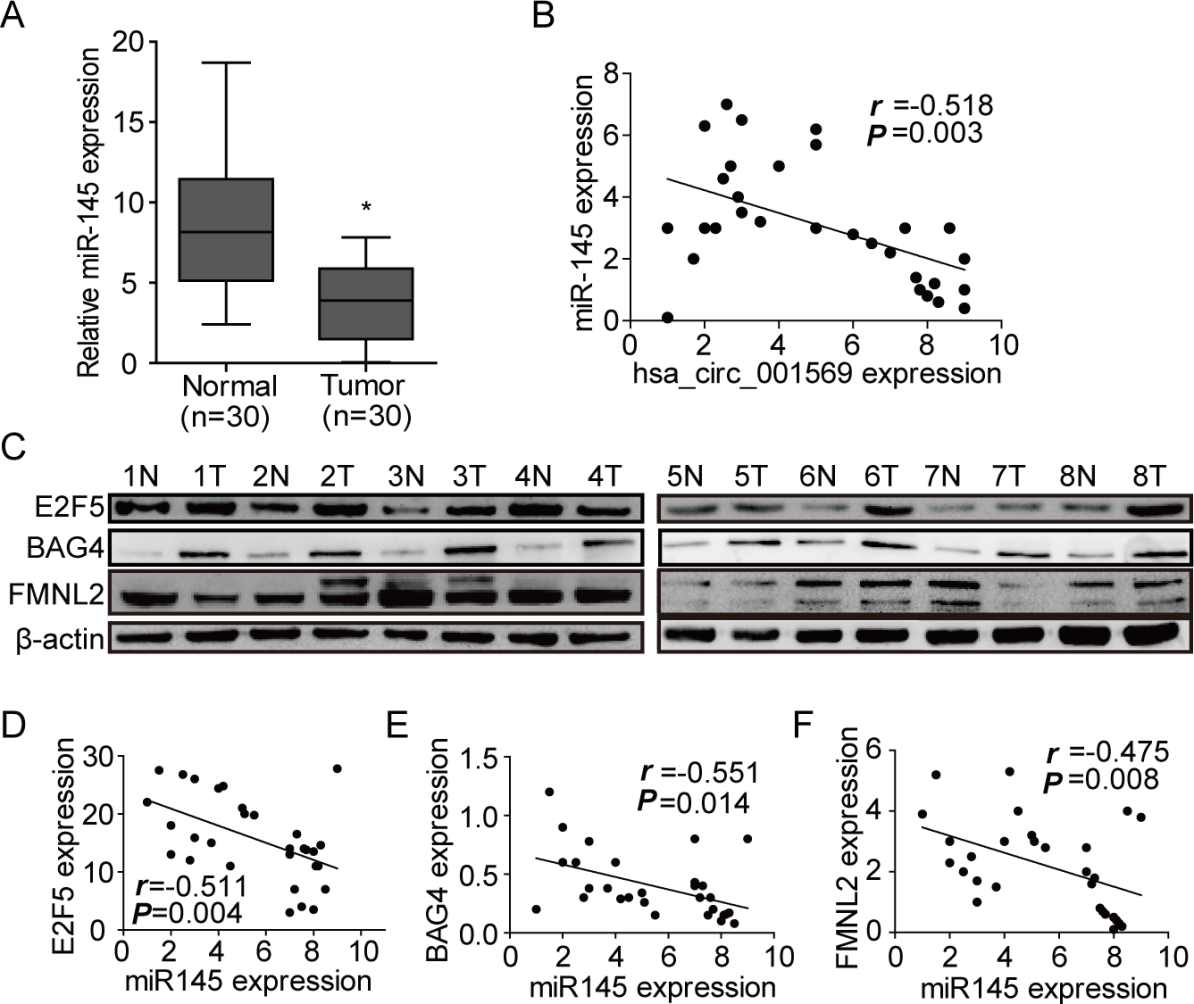
MiR-145 negatively correlates with circ_001569 or its targets (**A**) Expressions of miR-145 in 30 paired human CRC tissues and the corresponding normal mucosa by real-time PCR. *p < 0.05. (**B**) Expression correlation between hsa_circ_001569 and miR-145. (**C**) Expressions of E2F5, BAG4 and FMNL2 in 30 paired human CRC tissues and the corresponding normal mucosa by Western blotting. (**D–F**) Expression correlations between miR-145 and E2F5, BAG4 or FMNL2.

To further examine the relationship between circ_001569 and miR-145, miR-145 and its targets, we detected the expressions of circ_001569, miR-145 and its targets in the same 30 cases of fresh paired CRC tissues by real-time RT-PCR or Western blotting. Results showed that the level of miR-145 was significantly lower in the CRC tissues than adjacent normal tissues (*p* < 0.05, Figure 6A). Circ_001569 expression was higher in CRC tissues than adjacent normal tissues (Figure 1A). Circ_001569 expression negatively correlated with miR-145 expression in CRC tissues (*r* = −0.518, *p* = 0.003, Figure 6B). The protein levels of E2F5, BAG4 and FMNL2 were dramatically up-regulated in CRC tissue samples (Figure 6C). MiR-145 negatively correlated with the expressions of E2F5 (*r* = −0.511, *p* = 0.004, Figure 6D), BAG4 (*r* = −0.551, *p* = 0.014, Figure 6E), and FMNL2 (*r* = −0.475, *p* = 0.008, Figure 6F), respectively. Taken together, it can be concluded that reduced miR-145 expression by circ_001569 along with increased E2F5, BAG4 and FMNL2 expression are frequent events in human CRC cells.

## DISCUSSION

CircRNAs are widely expressed in human cells, and their expression levels can be 10-fold or higher compared to their linear isomers [25]. The two most important properties of circRNAs are highly conserved sequences and a high degree of stability in mammalian cells [8]. Compared with other noncoding RNA, such as miRNAs and longnoncoding RNAs (lncRNAs), these properties provide circRNAs with the potential to become ideal biomarkers in the diagnosis of cancers. There are only 12 researches about the relationship between circRNA and tumour. A negative correlation of global reduction circular RNA abundance and proliferation is observed in CRC [21], hsa_circ_002059 can be used as a biomarker for gastric cancer [26]. Hundreds of circRNAs are regulated during human epithelial-mesenchymal transition (EMT) [27]. Circular RNA ITCH has inhibitory effect on esophageal squamous cell carcinoma by suppressing the Wnt/β-catenin pathway [28]. Therefore, the clear function relationship between circular RNA and cancer as well as the regulatory mechanisms need to be further explored.

In this study, based on the previous study [21] and analyses on circ2Traits [22] public databases, we found that the level of circ_001569 was significantly higher in CRC tissues than adjacent normal tissues. Circ_001569 promoted CRC cell proliferation, at least partly by eliminating cell cycle arrest and inhibiting apoptosis. Circ_001569 also increased the invasive abilities of CRC cells. Circ_001569 is located on chromosome 16q13.1 on the plus strand, and aligned in a sense orientation to the known protein-coding gene ABCC1, and spans exons 33. Till now, the function of circ_001569 in tumor development and progression is unknown understood. Emerging data show that there are some potential effects between circRNAs and miRNAs, but the interactions of circRNAs with miRNAs on the progression of CRC remain largely elusive. According to the prediction results of StarBase v2.0 [23] and circBase [24], we selected miR-145 that might interact with circ_001569. Luciferase activity assays further validated that hsa_circ_001569 inhibited the transcription activity of miR-145 in CRC cells. The exonic circRNAs of *CDR1as* and *SRY* have been shown to bind miRNAs without being degraded, making them excellent candidates for competing endogenous RNA activity [29]. Similarly, we found that circ_001569 did not affect the expression of miR-145. This suggests that as a sponge of miR-145, circ_001569 may competitively bind and inhibit miR-145 activity, resulting in increased levels of miR-145 targets.

MiR-145 has been found to be deregulated in several tumors such as hepatocellular cancer, esophageal cancer, lung cancer, breast cancer [30–33]. MiR-145 is also closely related with the development of colon cancer [19, 20]. In addition, transcriptional and post-transcriptional regulations play an important role in miR-145 expression in cancers [34–36]. Here, we showed that as the sponge of miR-145, circ_001569 inhibited the transcription activity of miR-145 and significantly promoted cell proliferation and invasion in CRC by inhibiting miR-145.

By using three common bioinformatic algorithms (TargetScan, Pictar and miRANDA), we chose E2F5, BAG4 and FMNL2 as the targets of miR-145. Previous studies have been shown that E2F5 is a member of the E2F transcription factor family that binds to the promoters of the target genes involved in cell cycle control [37]; BAG4 has been reported to be with aggressiveness of several cancers, such as ovarian cancer, breast cancer, pancreatic cancer [38, 39]; FMNL2 promotes CRC cells proliferation, motility, invasion, metastasis and epithelial-mesenchymal transition [11, 40]. Our results showed that E2F5, BAG4 or FMNL2 was not only responsible for cell proliferation and invasion induced by circ_001569, but also functional targets of miR-145. Thus, circ_001569 acts as a miRNA sponge to directly combine with miR-145, and subsequently up-regulates miR-145 targets E2F5, BAG4 and FMNL2 to exert its tumor promoting function in CRC cells.

Finally, we analyzed the relationship between circ_001569 and miR-145, miR-145 and its targets. Results showed that circ_001569, E2F5, BAG4 or FMNL2 expression was up-regulated in CRC tissues. There were negative correlations between circ_001569 and miR-145 (*p* < 0.05), miR-145 and its targets (*p* < 0.05). This evidence clearly validates the role of circ_001569/miR-145/targets axis in the progression of CRC.

In summary, our study indicates that circ_001569 is up-regulated in CRC tissues and promotes the proliferation and invasion of CRC. It acts as a sponge to directly inhibit miR-145 transcription, and subsequently affects the functions of miR-145 targets E2F5, BAG4 and FMNL2 in CRC cells. These findings reveal a new mechanistic connection between miR-145 and hsa_circ_001569 in regulating the progression of CRC and may provide new insights and therapeutic strategies for CRC prevention and treatment.

## MATERIALS AND METHODS

### Ethics statement

Investigation has been conducted in accordance with the ethical standards and according to the Declaration of Helsinki and according to national and international guidelines and has been approved by the authors’ institutional review board.

I promise that the study was performed according to the international, national and institutional rules considering animal experiments, clinical studies and biodiversity rights. The study protocol was approved by the animal ethics committee of Southern Medical University.

### Cell culture

Human CRC cell lines were purchased from Cell Bank of Type Culture Collection (Shanghai City, China). The cells were cultured in RMPI 1640 (Hyclone, USA), supplemented with 10% (v/v) fetal bovine serum (FBS). The cells were kept in an incubator under 5% CO_2_ at 37°C.

### Patient samples

A total of 30 CRC tissue samples and matched non-tumor normal tissue samples were obtained from patients who underwent surgical resection without prior radiotherapy and chemotherapy at the Department of General Surgery in Nanfang Hospital (Guangzhou, Guangdong Province, People's Republic of China). The specimens were immediately snap-frozen and stored at −80°C in an ultra-low temperature refrigerator until further analysis. Informed consent was taken from all subjects and this study was approved by the ethics committee of Southern Medical University.

### Cell transfection with siRNAs and plasmids

All the primers for has-circ_001569 and miR-145 detection assays were purchased from Ribobio. Transfection of has-circ_001569 siRNA(circ-in, 5′-GCATCGTGCAGGACTGGAA-3′) and negative control (NC) via hsa- circ_001569 siRNA kit (RIBOBIO, Guangzhou, China) according to the manufacturer's protocol. The primers used were as follows: si-has-circ_001569 sense, 5′ GCAUCGUGCAGGACUGGAAdTdT 3′ and antisense, 3′ dTdTCGUAGCACGUCCUGACCUU 5′. Human hsa_circ_001569 cDNA was synthesized and cloned into pLVX-IRESneo by GeneCopoeia (Guangzhou, China). By following Hansen TB's [41] procedures, an 1182 bp DNA fragment corresponding to exons 33 of the ABCC1 gene were used, and then 1 kb upstream and 200 bp downstream were added to the nonlinear splice sites. Also, an 800-bp DNA stretch was added to upstream of the splice acceptor site and inserted into downstream in the reverse orientation.

MiR-145 negative control, inhibitor (5′-ACGGAUU CCUGGGAAAACUGGAC-3′) and mimics (5′- GTCCA GTTTTCCCAGGAATCCCT -3′) were designed and synthesized by Ribobio (Guangzhou, China). MiR-145 lentivirus-expressing vector pEZX-MR01/miR-145 containing the enhanced green fluorescent protein (EGFP) gene (GeneCopoeia, Guangzhou, China) was transfected into lentiviral packaging cell lines 293T. Then 1 mL of viral supernatant containing 4 Attogram (Ag) of polybrene was added into CRC cell lines for stable transduction. After 14 days, puromycin resistant cell pools were established.

The coding DNA sequences (CDS) of E2F5, BAG4 and FMNL2 were amplified by PCR and transduced into vector pGC FU-GFP-LV respectively were purchased from GeneCopoeia (Guangzhou, China). To obtain the co-expressing cells, 2 μg/mL of E2F5, BAG4 and FMNL2 expressing vectors were then added into circ_001569 or miR-145 expressing cells, respectively. After 72 h, Western blotting was performed to detect the expression of E2F5, BAG4 or FMNL2. Cells were transfected with miR-145 inhibitor or negative control (NC) by using Lipofectamine 2000 (Invitrogen, Foster city, CA). The 3′-untranslated regions (UTRs) of E2F5, BAG4 and FMNL2 genes clone and their mutant fragments and an empty vector control, pEZX-MT01, referred to as luciferase control, were purchased from GeneCopoeia (Guangzhou, China).

### Luciferase activity assays

Cells were co-transfected with 3′UTR of E2F5, BAG4 and FMNL2 plasmids and their mutant fragments and miR-145 mimics by using Lipofectamine 2000 (Invitrogen, Foster city, CA) according to the manufacturer's protocol. Firefly and Renilla luciferase activities were measured consecutively by using Dual-Luciferase Reporter Assay System (Promega, Massachusetts, USA) after transfected for 48 h. Each assay was repeated in 6 independent experiments.

### Real-time RT-PCR

Total RNA was extracted from the cells and tissues using Trizol reagent (Invitrogen, USA) according to the manufacturer's protocol. For circRNA hsa_circ_001569, The RNase R digestion reaction was performed following Danan M's [42] procedures. The digestion and precipitation reactions were repeated twice with a ratio of 3 U enzyme/1 mg RNA. Then cDNAs were synthesized from total RNA using Prime-Script RT reagent kit (TaKaRa, Japan). The primers used in q-PCR were shown in Table S1. GAPDH was an internal control. The data were analyzed using the ΔCt method. All the primers were synthesized by Intrivogen. All results are expressed as the mean ± SD of three independent experiments.

### Western blotting

The Western blotting was performed according to established protocols [23], using anti-E2F5 (1:500, Abcam, Cambridge, MA, USA) and anti-BAG4 monoclonal antibody (1:500, Abcam, Cambridge, MA, USA), anti-FMNL2 monoclonal antibody (1:200, Abnova, Taibei City, Taiwan). An anti-α-tubulin (1:1000, Sigma, St. Louis, MO, USA) and anti-β-actin(1:1000, Sigma, St. Louis, MO, USA) monoclonal antibody was used as a loading control.

### Proliferation, cell cycle, apoptosis and cell invasion assays *in vitro* and *in vivo* tumorigenicity assay

The proliferation, cell cycle, apoptosis and invasion of transfected CRC cells [43], and *in vivo* tumorigenicity assay [11] were determined as previously described.

### Statistical analyses

All statistical analyses were performed using SPSS19.0 for Windows. The CCK-8 method and *in vitro* invasion assay were tested using one-way analysis of variance for factorial design. A paired *t* test was used to investigate the difference of the miR-145 or hsa_circ_001569 expression level between normal and cancerous tissues. A two-sample *t* test was used to analyze the clinicopathologic characteristics of miR-145 expression in CRC patients. The Pearson correlation coefficient was used to measure the degree of the linear relationship between the expression levels of miR-145 and E2F5, BAG4 or FMNL2; circ_001569 and miR-145 in CRC cells and tissues. Data were presented as the mean with 95% confidence intervals of at least 3 independent experiments. A *p* value less than .05 was considered statistically significant.

## SUPPLEMENTARY MATERIALS FIGURES AND TABLES


