# Supplementary material for: Emerging roles of circRNA_001569 targeting miR-145 in the proliferation and invasion of colorectal cancer
Source: Oncotarget. 2016 Apr 5;7(18):26680–91. doi: 10.18632/oncotarget.8589 (PMC5042007; doi:10.18632/oncotarget.8589)
Supplement: Supplementary file 1 [file oncotarget-07-26680-s001.pdf]

Emerging roles of circRNA_001569 targeting miR-145 in the proliferation and invasion of colorectal cancer

Supplementary Materials

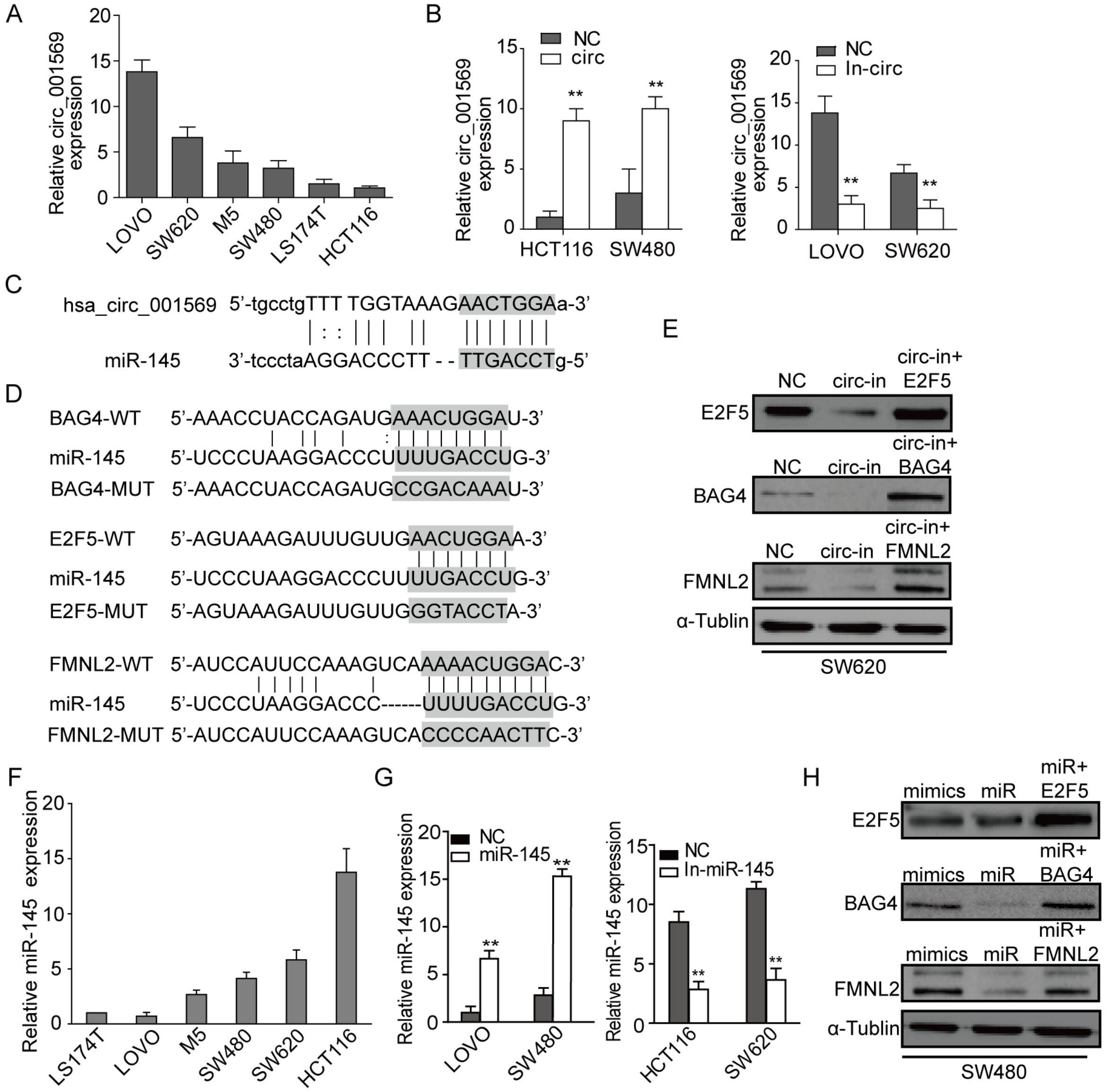

Supplementary Figure S1: The transfection efficiency of miR-145 and in CRC cells and the effect of hsa_circ_001569 on the expressions of miR-145 and its targets. (**A**) Endogenous expression of circ_001569 assessed by Real-time PCR in 6 CRC cell lines. (**B**) Expression of circ_001569 in cells transfected with circ_001569 expressing vector or circ_001569 inhibitor. (**C**) The binding sites of miR-145 to circ_001569 predicted by the starBase v2.0 website. (**D**) The binding sites of miR-145 to 3′UTRs and mutant fragments of E2F5, BAG4 or FMNL2 predicted by TargetScan, Pictar and miRANDA. (**E**) Western blot analyses of E2F5, BAG4 and FMNL2 in cells transfected with circ-in, circ-in/E2F5, circ-in/BAG4 and circ-in/FMNL2 in SW620 cells, respectively. (**F**) Endogenous expression of miR-145 assessed by Real-time PCR in 6 CRC cell lines. (**G**) Expression of miR-145 in cells transfected with miR-145 mimics or miR-145 inhibitor. (**H**) Western blot analyses of E2F5, BAG4 and FMNL2 in cells transfected with miR-145, miR-145/E2F5, miR-145/BAG4 and miR-145/FMNL2 in SW480 cells, respectively. Error bars represent mean ± SD from three independent experiments. ** $p < 0.01$.

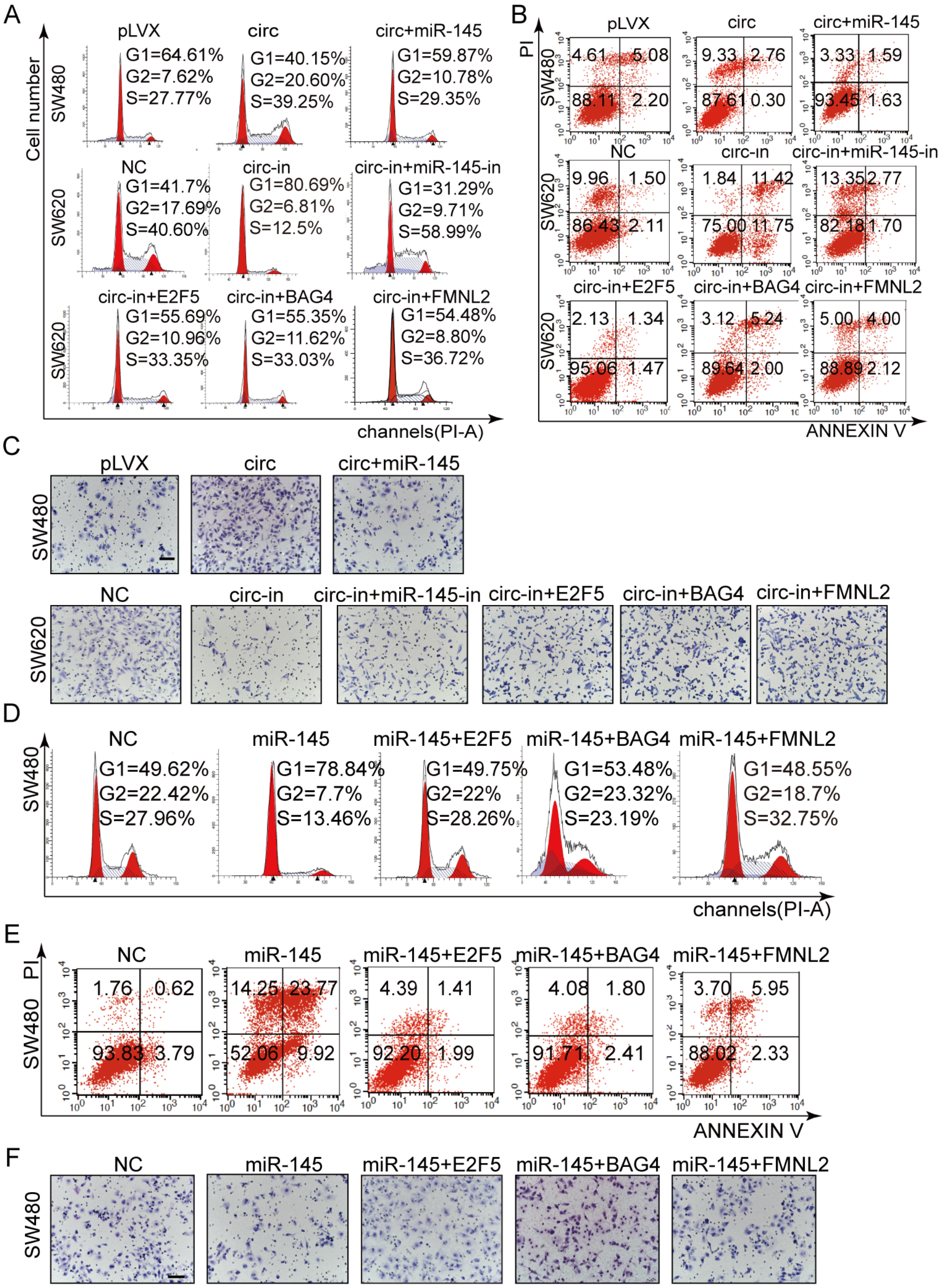

Supplementary Figure S2: Circ_001569 promotes cell proliferation and invasion by regulating miR-145 and its targets (**A**) Effect of miR-145, E2F5, BAG4 or FMNL2 on circ_001569 induced CRC cell cycle by flow cytometry. (**B**) Effect of miR-145, E2F5, BAG4 or FMNL2 on circ_001569 induced CRC cell apoptosis by flow cytometry. (**C**) Effect of miR-145, E2F5, BAG4 or FMNL2 on circ_001569 induced cell invasion *in vitro* by Boyden chamber. (**D**) Effect of E2F5, BAG4 or FMNL2 on miR-145 induced CRC cell cycle by flow cytometry. (**E**) Effect of E2F5, BAG4 or FMNL2 on miR-145 induced CRC cell apoptosis by flow cytometry. (**F**) Effect of E2F5, BAG4 or FMNL2 on miR-145 induced cell invasion *in vitro* by Boyden chamber.

Supplementary Table S1: Primer sequences used for quantitative real-time PCR. (5′ to 3′)

Gene	Forward primer	Reverse primer
miR-145	GTCCAGTTTTCCCAGGAATCCCT	CCCAGGAAUCCCU
E2F5	GGGCTGCTCACTACCAAGTTC	CCTACACCTTTCCACTGGATACT
BAG4	AATGGAGCGTATGGTCCAACA	GGTGCATAATAAGCCCCTGAGT
FMNL2	CAGGGAGCATGGATTCGCAG	TCAGGAGGTAGGTTCATAGCATT
hsa_circ_001569	TCCCCTGAACATTCTCCCCAT	GAAAGCACTTGGTGAAGTCGG
GAPDH	CACCGTAGCCTTCCGAGTA	GCCCTTGATGAGCTGTTGA
